# Effects of Ultrasonic-Assisted Enzymatic Treatment on the Solubility and Stability of Myofibrillar Protein from Tilapia (*Oreochromis niloticus*)

**DOI:** 10.3390/foods14244232

**Published:** 2025-12-09

**Authors:** Juanjuan Zhao, Huan Xiang, Hui Huang, Ya Wei, Yongqiang Zhao, Shuxian Hao

**Affiliations:** 1 College of Food Science and Technology, Shanghai Ocean University, Shanghai 201306, China; zhaojuanjuan0222@163.com; 2 Key Laboratory of Aquatic Product Processing, Ministry of Agriculture and Rural Affairs, National R&D Center for Aquatic Product Processing, South China Sea Fisheries Research Institute, Chinese Academy of Fishery Sciences, Guangzhou 510300, Chinazhaoyq@scsfri.ac.cn (Y.Z.); 3 Key Laboratory of Efficient Utilization and Processing of Marine Fishery Resources of Hainan Province, Sanya Tropical Fisheries Research Institute, Sanya 572018, China

**Keywords:** ultrasonic-assisted enzymatic treatment, myofibrillar protein, solubility in low-salt solution

## Abstract

Myofibrillar protein (MP) aggregation in solutions with NaCl concentrations below 0.3 M results in poor solubility. Ultrasound-assisted glutaminase treatment (UGT) was applied to improve MP solubility in a low-salt solution (containing 0.1 M NaCl). The solubility increased with ultrasonic power and time, peaking at 44.34% (480 W, 15 min) and reaching 61% after UGT. Subsequently, the effect of post-sonication heat treatment (60 °C, 30 min) on the physicochemical and structural characteristics of ultrasound-enzyme treated MP (UEMP), prepared under specific ultrasonic conditions (480 W, 20 min), was systematically investigated. The findings revealed that UEMP exhibited higher hydrophobicity, sulfhydryl content, and turbidity, but reduced particle size, ζ-potential, and fluorescence, suggesting disulfide disruption and exposure of hydrophobic residues. Sodium dodecyl sulfate-polyacrylamide gel electrophoresis revealed weakened high-molecular weight bands and intensified low-molecular weight bands. Fourier-transform infrared spectroscopy confirmed these structural rearrangements, with a blue-shifted amide A band and decreased amide I intensity. Heating further increased the hydrophobicity and fluorescence without altering the size, ζ-potential, or molecular weight. The red shift in the amide A band suggests reinforced local ordering. Rheology analysis showed non-Newtonian shear-thinning behavior, which was unchanged by UGT or heating. Collectively, UGT with moderate heating enhances MP solubility and thermal stability by disrupting stabilizing bonds and modulating the structure.

## 1. Introduction

Currently, China is the world’s leading producer of farmed tilapia (*Oreochromis mossambicus*), making it the country’s most important aquatic species for export [1]. This status can be attributed partly to tilapia’s rapid growth and strong adaptability and partly to its desirable flesh properties, including delicate taste, few intermuscular bones, and high nutritional value, which have made it widely favored by consumers [2,3]. Myofibrillar protein (MP) is an important component of muscle, consisting of thick and thin filaments fixed by M protein, titin, and α-actinin. Thick filaments are composed of myosin, whereas thin filaments are composed of C-proteins and actin [4]. Studies have shown that, unlike the aggregation behavior of MP in solutions with NaCl concentrations below 0.3 M, this protein is highly soluble in 2–3% NaCl solutions, equivalent to 0.47–0.68 M NaCl solutions [5], providing insights into the processing of meat protein products. Currently, meat protein products mainly include preserved meat [6], meat emulsion gels [7], and emulsified foods [8]. However, long-term high salt intake increases the risk of developing cardiovascular diseases, hypertension [9,10], and osteoporosis [11].

To stabilize and improve the quality of meat protein products under low-salt conditions, researchers have studied the effects of exogenous substances, such as tripolyphosphate, plant polysaccharides, enzymes, and phenolic compounds, as well as cutting-edge technologies, such as ultrasound, high pressure, and plasma jet, on the structure and function of MP [12]. Ultrasound, a low-temperature processing technology, generates numerous cavitation bubbles that collapse, releasing heat energy and strong shear forces, thereby disrupting protein–protein interactions. This technique has been applied to chicken [13], egg white protein [14], and squid [15], leading to enhanced functional properties. Enzymatic treatment has attracted widespread attention for protein modification owing to its efficiency, safety, mild reaction conditions, high reaction speed, and substrate specificity [16,17]. With the advancement of related studies, combined treatments have generally been shown to outperform single treatments in enhancing the structural and functional properties of MP. For instance, Chen et al. [18] reported that ultrasound-assisted conjugation of epicatechin gallate with chicken myofibrillar proteins markedly enhanced both antioxidant capacity and in vitro digestibility compared with treatment with epicatechin gallate alone. And the study of Liu et al. [19] has shown that under the combined action of ultrasound and glutaminase, the zeta potential and particle size of chicken myofibrillar proteins are significantly reduced, promoting the solubility of myofibrillar proteins in low-salt solutions. However, Amiri et al. [20] reported that ultrasound-assisted papain treatment can induce further aggregation of MPs, leading to decreased solubility, water-holding capacity, and surface hydrophobicity, accompanied by a shift in particle size distribution toward larger aggregates and increased turbidity.

To expand the application of ultrasound-enzyme combination treatment in protein modification and promote the development of novel animal protein-based products, tilapia myofibrillar protein (TMP) was selected as the substrate in this study. TMP samples were prepared under varying ultrasonic intensities and durations in combination with glutaminase treatments. Furthermore, heat treatment (60 °C, 30 min) and enzyme treatment were applied in succession to the samples treated under specific ultrasonic conditions (480 W, 20 min). The physicochemical properties of and structural changes in the myofibrillar protein were systematically characterized to clarify the effects of ultrasonic-assisted enzymatic treatment on its solubility and thermal stability.

## 2. Materials and Methods

### 2.1. Materials and Reagents

Tilapia was purchased from a supermarket (Guangzhou, Guangdong, China). Glutaminase (EC3.5.1.2) was purchased from Amano Company (Yokohama, Japan). Disodium phosphate dodecahydrate and dihydrogen phosphate dihydrate was purchased from Shanghai Macklin Biochemical Co., Ltd. (Shanghai, China). SDS-PAGE Protein Sample Buffer (5×), BeyoBlue™ Coomassie Blue Ultra-Fast Staining Solution, SDS-PAGE Electrophoresis Buffer (Tris-Gly, 10×), and Coomassie Blue stain were purchased from Shanghai Beyotime Biotechnology Co., Ltd. (Shanghai, China). Destaining solution was purchased from Shanghai Beyotime Biotechnology Co., Ltd. (P0017C).

### 2.2. Extraction of MP

Tilapia meat (50 g) was mixed with four volumes of phosphate-buffered saline (PBS, containing 12.4 mM Na_2_HPO_4_·12H_2_O and 6.6 mM NaH_2_PO_4_·2H_2_O, pH 7.0; 200 mL). The mixture was homogenized using a high-speed dispenser (T25 Digital, Staufen, Germany) for 1 min and centrifuged for 10 min at 10,000× *g* using a high-speed desktop refrigerated centrifuge (H1850R, Changsha, China). The supernatant was discarded, and the process was repeated for the precipitates. The final precipitate was mixed with four volumes of high-salt PBS (0.6 mol/L NaCl-0.02 mol/L PBS, pH 7.0) and homogenized. After homogenization, the solution was allowed to stand at 4 °C for 30 min for extraction and then centrifuged at 4 °C and 10,000× *g* for 10 min. The myofibrillar protein (MP) solution, whose concentration was determined using the Biuret method, was obtained by collecting the supernatant. Finally, the MP solution was frozen at −40 °C for 12 h before being placed in a freeze dryer (SCIENTZ-30FG/A, Ningbo, China) for freeze-drying and storage for future use.

### 2.3. Ultrasonic Treatment of MP

MP powder (1 g) was dissolved in 100 mL of low-salt PBS (0.02 mol/L, containing 0.1 mol/L NaCl, pH 7.0) to yield a suspension with a final concentration of 10 mg/mL. The suspension was vortexed for 10 min and transferred to a thermostatic vessel for ultrasonic treatment using a cell disruptor (JY99-IIDN, Shanghai, China). Sonication was conducted at output powers of 120, 240, 360, and 480 W for total durations of 0, 5, 10, 15, and 20 min, following the method described by Wu et al. [21] with minor modifications. Throughout the process, the sample temperature was maintained at ≤4 °C under pulsed conditions (2 s on, 4 s off). The ultrasonic probe (22 mm diameter) was immersed 1.5 cm above the bottom of the vessel to ensure uniform energy distribution. After sonication, all samples were incubated at 60 °C for 30 min to enable comparison under consistent thermal conditions. Following cooling to 25 °C, the protein concentration was determined using the biuret method. The samples were designated as 120 UMP, 240 UMP, 360 UMP, and 480 UMP and stored at 4 °C until further analysis.

### 2.4. Enzymatic Treatment of MP

The pH of 100 mL of a 10 mg/mL ultrasound-treated MP solution was adjusted to 7.0 using 3 M sodium hydroxide or hydrochloric acid solution. Glutaminase was added to the solution at a ratio of 1:200 (enzyme: protein, *w*/*w*) by weight, and the reaction was carried out at 50 °C for 4 h in a water bath thermostatic oscillator (SHZ-82A, Chang Zhou, China). The enzyme was inactivated by boiling the solution in a water bath for 10 min, after which the solution was cooled to 25 °C with running water and stored in a refrigerator at 4 °C for later use. The samples treated with ultrasound and enzyme treatment were named 120 UEMP, 240 UEMP, 360 UEMP, and 480 UEMP.

### 2.5. Determination of Solubility

A 1 mL volume of bovine serum albumin standard solution (0, 1, 2, 3, 4, 5 mg/mL) was mixed with 4 mL of biuret reagent and reacted in the dark for 30 min, after which the absorbance of the mixture is measured. Then, a standard curve (Y = 0.0255X + 0.0363, R^2^ = 0.999) was plotted with the concentration of bovine serum albumin standard solution as the *x*-axis and the absorbance of each mixture as the *y*-axis. The samples were centrifuged at 10,000× *g* and 4 °C for 10 min, and the supernatants were separated into 10 mL tubes for subsequent use. Then, 1 mL of the supernatant was taken, 4 mL of biuret reagent was added, and the mixture was allowed to react in the dark for 30 min. A microplate reader (Varioskan L U X, Waltham, MA, USA) was used to measure the absorbance, calculate the average value, and substitute it into the standard curve to obtain the protein concentration in the supernatant. The solubility of MP was calculated using Equation (1):

(1) $$\mathrm{Solubility}\left(\%\right)=\frac{C_{S}}{C_{0}}\times 100$$

where *C_S_* and *C*_0_ represent the protein concentrations in the supernatant of the sample [mg/mL] and the original protein concentration [mg/mL], respectively.

### 2.6. Physicochemical Properties of MP

#### 2.6.1. Determination of Surface Hydrophobicity

This was performed according to the method described by Li et al. [22] with slight modifications. MP solutions from the different treatments were diluted to a mass concentration of 1 mg/mL using PBS buffer (containing 12.4 mM Na_2_HPO_4_·12H_2_O, 6.6 mM NaH_2_PO_4_·2H_2_O, and 0.1 mol/L NaCl, pH 7.0). Each 2 mL sample was thoroughly mixed with 80 μL of 1 mg/mL bromophenol blue (BPB) solution, and PBS was used as a control. After incubation at 25 °C for 20 min, the samples were centrifuged at 4000× *g* for 15 min. After a 10-fold dilution, the absorbance of the supernatant was measured at 595 nm using a microplate reader. Surface hydrophobicity was expressed as the amount of BPB binding, which was calculated using Equation (2):

(2) $$\mathrm{BPB}\;\mathrm{binding}(\mu g)=80\times \frac{\left(A_{0}-A_{S}\right)}{A_{0}}$$

where *A*_0_ and *A_S_* represent the absorbance of the control and sample groups, respectively.

#### 2.6.2. Determination of Total Sulfhydryl Content

This was performed according to the method described by Shen, Tang and Li [23], with slight modifications. To determine the total sulfhydryl content, 300 μL of a 1 mg/mL MP solution (pH 7.0) was added to 2.7 mL of PBS (containing 10 mmol/L Na_2_HPO_4_/NaH_2_PO_4_, 0.15 mol/L NaCl, 8 mol/L urea, pH 7.0). Then, 400 μL of 10 mmol/L 2-nitrobenzoic acid solution was added to the well-mixed solution. The mixture was vortexed and incubated at 37 °C in the dark for 30 min. PBS was used as the control, and the absorbance of the samples was measured at 412 nm wavelength. The total sulfhydryl content was calculated using Equation (3):

(3) $$C_{T}=D\times 10^{6}\times \frac{A}{\varepsilon }$$

where C*_T_* represents the total sulfhydryl content (μmol/g), *A* represents the absorbance of the sample at 412 nm, *ε* represents the molar absorptivity (13,600 L/(mol·cm)), and D is the dilution factor (10).

#### 2.6.3. Determination of Turbidity

The turbidity of the MP solutions was assayed using the method described by Li et al. [22] with slight modifications. Each sample was diluted to 1.0 mg/mL using 0.02 M PBS, and the absorbance was measured at 600 nm using a UV-Visible spectrophotometer (Lambda 365+, Waltham, MA, USA) with deionized water as the blank. Absorbance was expressed as turbidity.

#### 2.6.4. SDS-PAGE

An appropriate amount of MP solution was mixed with protein loading buffer (5×) in a 4:1 ratio, mixed well, heated in a boiling water bath for 5 min, and centrifuged for 2 min. The supernatant was collected for loading electrophoresis (the acrylamide concentration in the SDS-PAGE gel was 12%) using an electrophoresis apparatus (Mini-PROTEAN Tetra/PowerPac, Hercules, CA, USA) at 80 V for 30 min before increasing the voltage to 100 V. After electrophoresis was complete, the gel was stained with BeyoBlue™ Coomassie Blue Ultra-Fast Staining Solution. After complete staining, the staining solution was removed, and an appropriate amount of destaining solution was added. The gel was then destained for 12 h and scanned using a gel imaging system (Tanon MINI SPACE 3000, Shanghai, China).

#### 2.6.5. Size and Zeta Potential

This was performed according to the method described by Xiang et al. [24] with minor modifications. The samples were appropriately diluted with 0.02 M PBS and filtered using a 0.45 μm filter membrane (the filtration step was omitted when measuring zeta potential). The size and zeta potential were measured using a laser particle size analyzer (Zetasizer Nano ZS90, Malvern, Worcestershire, UK). Note that when loading the samples, they were injected slowly to avoid the formation of bubbles, and the measurement was performed three times to reduce errors.

### 2.7. Structural Characteristics

#### 2.7.1. Fourier Transform Infrared Spectroscopy (FTIR) Analysis

An appropriate amount of freeze-dried sample was collected and mixed with potassium bromide. The mixture was ground, and a tablet press was used to form pellets. Secondary structural changes in the MP samples were detected using a Fourier transform infrared spectrometer (IRAffinity-1, Kyoto, Japan). The parameters were set as follows: scan range from 4000 to 400 cm^−1^ and scan rate of 64 scans per second.

#### 2.7.2. Intrinsic Fluorescence Spectroscopy

The samples were diluted with 20 mM PBS (containing 1.0 mM EDTA, pH 7.5) to 1.0 mg/mL. Fluorescence spectroscopy scans were performed using a microplate reader with an excitation wavelength of 280 nm and a scanning range of 300–450 nm. The fluorescence intensity was measured at the maximum emission wavelength [25].

### 2.8. Determination of Rheological Properties

Samples were diluted to a final concentration of 1 mg/mL in 20 mmol/L PBS supplemented with 0.1 mol/L NaCl (pH 7.0). The apparent viscosity of the suspensions was measured using a rotational rheometer (MCR 502, Anton Paar, Austria) across a shear rate range of 1–100 s^−1^, following the procedure outlined by Sağlam et al. [26].

### 2.9. Statistical Analysis

Statistical analyses were performed using SPSS software (IBM SPSS Statistics 27) for correlation and significance analyses, and Origin 2021 software was used for graphing the results. Differences among the treatment groups were analyzed using one-way analysis of variance (ANOVA). Unless otherwise specified, data are presented as mean ± standard deviation (SD), and statistical significance was set at *p* < 0.05.

## 3. Results and Analysis

### 3.1. Solubility

**Figure 1:**
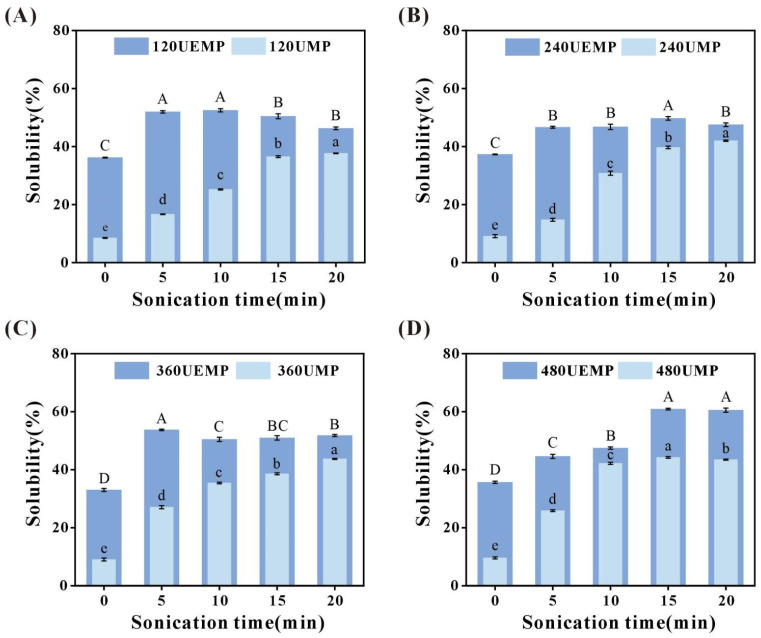
Changes in the solubility of myofibrillar protein within 0~20 min under different intensities of ultrasound: (**A**) 120 W, (**B**) 240 W, (**C**) 360 W, and (**D**) 480 W. (UEMP: ultrasound-enzyme-treated myofibrillar protein; UMP: ultrasound-treated myofibrillar protein). Note: Uppercase and lowercase letters indicate significant differences in solubility of UEMP/UMP at different sonication times, respectively.

As shown in Figure 1, the solubility of untreated MP was 8.9%, indicating its poor solubility in low-salt phosphate-buffered saline (PBS). However, the solubility of UMP significantly increased in a time- and power-dependent manner, reaching a maximum of 44.34% after treatment at 480 W for 15 min. This phenomenon may be attributed to the capacity of ultrasound to disrupt protein aggregates, inducing their dissociation and dispersion [27]. This structural disintegration allows a greater proportion of protein molecules to remain in a dispersed state within the solution, ultimately enhancing their solubility. However, prolonging the duration of ultrasound exposure to 20 min at the same output power markedly reduced protein solubility, indicating that excessive treatment has detrimental effects. This observation is consistent with the findings of Hong et al. [28], who reported that high-intensity ultrasound promotes the formation of insoluble protein aggregates. In addition, UGT for 0–20 min substantially enhanced UMP solubility, with the minimum value rising from 42.02% to 47.52% (from 240 UMP to 240 UEMP at 20 min) and the maximum increasing from 16.68% to 51.96% (from 120 UMP to 120 UEMP at 5 min). This enhancement is likely attributable to ultrasound-induced conformational unfolding of MP, which exposes glutamine (Gln) residues that serve as catalytic sites for glutaminase. Exposure of these residues facilitates their deamidation to glutamic acid (Glu), thereby increasing the overall negative charge of the protein molecules. This charge alteration strengthens the electrostatic repulsion between MP molecules and ultimately enhances their solubility [29]. Notably, the 480 UEMP group (ultrasound for 15 and 20 min) exhibited the highest solubility of 60.88%, and no significant change was observed when the ultrasonic time was extended to 20 min under the same conditions.

In summary, the solubility of MP reached its maximum value after 20 min of 480 W ultrasonic and enzymatic treatment. Based on these findings, subsequent experiments were conducted to investigate the effects of heating and non-heating treatments on the physicochemical properties, structure, and viscosity of myofibrillar protein subjected to 480 W ultrasonic treatment for 20 min, with or without enzymatic treatment. Untreated myofibrillar protein is denoted as MP, the glutaminase-treated myofibrillar protein as EMP, ultrasound-treated myofibrillar protein as UMP, ultrasound- and enzyme-treated myofibrillar protein as UEMP, and the corresponding heat-treated myofibrillar proteins are denoted as HMP, HEMP, HUMP, and HUEMP.

### 3.2. Physicochemical Properties

#### 3.2.1. Surface Hydrophobicity

As shown in Table 1, the surface hydrophobicity (H_0_) of MP significantly increased after enzymatic treatment, ultrasonic treatment, and UGT. Compared to ultrasonic treatment alone, enzymatic treatment and UGT significantly increased MP H0. This may be because both ultrasonic and enzymatic treatments promote MP depolymerization. While the cavitation effect of ultrasound opens up the structure of MP, glutaminase further binds to the protein, promoting MP uncoiling and exposing the internal hydrophobic groups of the protein. This result is similar to that of Amiri et al. [20], who believed that ultrasonic cavitation and enzymatic treatment can cause structural changes in proteins, leading to the migration of masked hydrophobic groups to the protein surface and an increase in H_0_ value. Additionally, the H_0_ of MP significantly increased after treatment at 60 °C for 30 min. Similar to untreated MP, the surface hydrophobicity of heat-treated MP increased significantly after ultrasonic treatment, enzymatic treatment, and UGT. This indicates that the structure of MP changes after ultrasonic, enzymatic, ultrasound-assisted enzymatic, and heat treatments, and that after heat treatment, the structure of MP may become more extended, exposing the internal hydrophobic groups on the protein surface. Han et al. [30] also showed that heating induces the unfolding and rearrangement of hydrophobic regions in MP, which can expose hydrophobic groups and further promote hydrophobic aggregation between protein molecules.

#### 3.2.2. Total Sulfhydryl Content

The total sulfhydryl content includes both free sulfhydryl groups and those within the protein that are not involved in disulfide bond formation. Changes in the total sulfhydryl content reflect the level of disulfide bonds; when the total sulfhydryl content decreases, it indicates that active sulfhydryl groups in the protein have combined to form these bonds. As shown in Table 1, the total sulfhydryl content of the untreated MP was 101.40 ± 8.61 μmol/g protein. Following enzyme treatment, a slight reduction in this value indicated a potential increase in disulfide bond levels. This outcome aligns with the findings of transglutaminase-treated myofibrillar proteins of *Penaeus vannamei* [31]. This effect is likely due to enzyme-induced conformational rearrangements that promote intermolecular disulfide bonding and facilitate the aggregation of MPs. In contrast, ultrasonic-assisted enzyme treatment increased the total sulfhydryl content to 112.75 ± 4.64 μmol/g of protein. A previous study demonstrated that the intense shear forces and turbulence generated by ultrasonic cavitation disrupt intra- and intermolecular bonds, thereby exposing more reactive sulfhydryl groups [32]. Furthermore, the difference in total sulfhydryl content between ultrasound alone and ultrasound-enzyme combined treatments may result from the ability of glutaminase to scavenge ultrasound-induced free radicals [33], thereby preventing the oxidation of sulfhydryl. Under heat treatment, the total sulfhydryl content of the HMP and HUEMP groups increased significantly, reaching 127.92 ± 22.79 and 129.88 ± 20.47 μmol/g of protein, respectively. This phenomenon is likely attributable to heat-induced protein denaturation and chain extension, which expose previously buried sulfhydryl groups to the solvent [34].

#### 3.2.3. Turbidity

**Figure 2:**
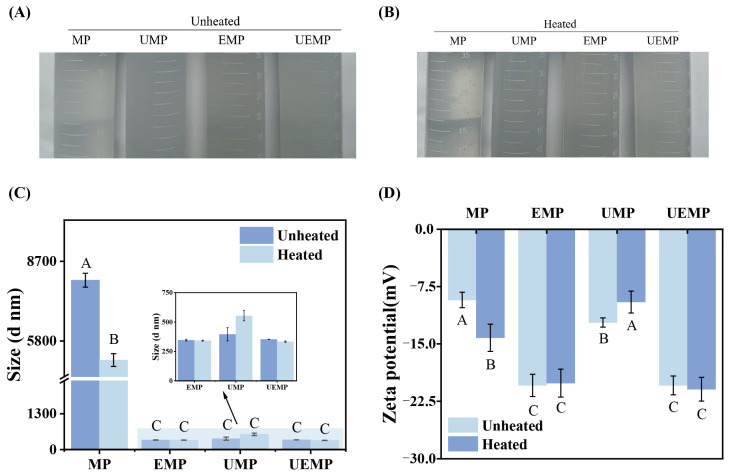
Physicochemical properties of samples: (**A**) Turbidity of unheated MP in control and treatment groups, (**B**) Turbidity of heated MP in control and treatment groups, (**C**) Size, (**D**) Zeta potential. (MP: control; EMP: enzymatically treated myofibrillar protein; UMP: ultrasound-treated myofibrillar protein; UEMP: ultrasound-enzyme-treated myofibrillar protein). Note: Uppercase letters indicate significant differences in particle size and ζ-potential among different treatment groups.

As shown in Table 1 and Figure 2A,B, the turbidity of the untreated MP solution was 0.153 ± 0.008. In contrast, both ultrasonic treatment and UGT markedly increased the turbidity. This enhancement is likely attributable to cavitation and microstreaming effects, which induce partial protein unfolding and secondary aggregation or dispersion, thereby altering the particle size distribution, enhancing light scattering, and promoting a more uniform dispersion of proteins in low-salt PBS. However, this trend contrasts with the findings of Xie et al. [35], who reported that turbidity decreased significantly in porcine MP solutions subjected to 300 W ultrasound treatment for 20 min. This difference may be due to differences in the MP type and ultrasonic conditions used. Dara et al. [36] noted that tuna MP aggregated more easily than MP from other livestock and poultry, with significantly higher turbidity. Conversely, turbidity decreased in all groups after heat treatment. Previous studies have similarly shown that during incubation at 50–80 °C, the turbidity of myosin solutions decreases progressively with increasing temperature [37]. This phenomenon likely reflects the competing effects of heat-induced protein unfolding and aggregation, in which conformational unfolding reduces light-scattering capacity, thereby lowering turbidity.

#### 3.2.4. Size and ζ-Potential

As shown in Figure 2C, native MP predominantly exists as large macromolecular aggregates, with particle sizes reaching approximately 8034.33 nm. However, upon modification, the particle size of MP was markedly reduced, with the HUEMP group displaying the smallest dimensions (333.87 nm). Compared to the control, ultrasound treatment, glutaminase treatment, and their combination significantly reduced MP particle size. This reduction is most likely attributable to the intense mechanical forces generated by acoustic cavitation, which disrupts electrostatic interactions, hydrogen bonding, and other noncovalent associations among MP molecules. Such disruption facilitates protein depolymerization, leading to the formation of smaller protein fragments, thereby enhancing molecular dispersion [38]. The regulatory influence of glutaminase on MP particle size is likely linked to its effect on protein charge distribution. A previous study reported that deamidation can neutralize positively charged clusters located at the C-terminal region of myosin, thereby disrupting the ordered assembly of myosin dimers and inhibiting the formation of thick filaments [39]. This mechanistic framework provides a coherent explanation for the reduction in particle size observed after ultrasound-enzymatic treatment. Moreover, apart from the reduction in MP particle size and the concomitant increase in UMP particle size observed after thermal treatment, no significant differences were detected in the other treatment groups before and after heating. These findings suggest that moderate heating can promote MP depolymerization, whereas it may accelerate the aggregation of MP pretreated with ultrasound but does not cause UEMP aggregation [40]. Consistent with these observations, Li et al. [1] showed that ultrasound combined with microwaves or other physical treatments effectively reduces MP particle size; however, when ultrasonic intensity exceeds a certain threshold, the particle size of co-treated samples increases substantially, highlighting the existence of a power-dependent threshold effect.

Figure 2D shows that before modification, the absolute value of the ζ-potential of the MP solution was relatively small, indicating an uneven particle distribution and poor stability. After modification, except for HUMP, which showed no significant change in the absolute value of the ζ-potential, the absolute values of all other treated groups significantly increased, with the EMP, UEMP, HEMP, and HUEMP groups showing the largest absolute ζ-potential values. Additionally, heat treatment had little effect on MP after enzymatic treatment or UGT. Therefore, we can conclude that heat treatment, ultrasound, enzymatic treatment, and UGT can all promote the dissolution of MP, which is also supported by the results mentioned above. The significant decrease in the absolute value of the ζ-potential in the heat-treated group after ultrasound exposure may be due to the thermal energy transferred to the MP during the cavitation bubble collapse during ultrasound exposure, which caused a vigorous molecular motion. Further heat treatment exacerbated this thermal motion, promoting the aggregation of MP into larger particles and burying negatively charged groups on the protein surface. This result is consistent with the particle size results. Sun et al. [41] found that ultrasound can enhance the absolute value of the ζ-potential of soy protein isolate, potato protein isolate, and soy/potato protein complexes while reducing their particle sizes. Additionally, studies have shown that ultrasound and enzymatic treatments can reduce the particle size of MP and increase the absolute value of the ζ-potential [42,43].

### 3.3. Analysis of Structural Characteristics

#### 3.3.1. Analysis of SDS-PAGE

**Figure 3:**
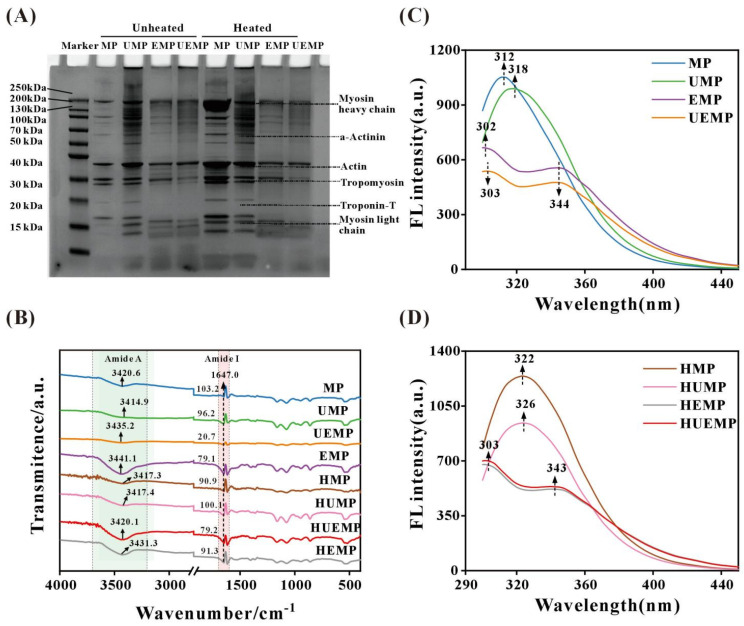
Structural characteristics of samples: (**A**) Sodium Dodecyl Sulfate-Polyacrylamide Gel Electrophoresis Pattern of samples, (**B**) Fourier Transform Infrared Spectroscopy of samples, (**C**) Fluorescence spectroscopy of unheated MP, (**D**) Fluorescence spectroscopy of heated MP. MP, EMP, UMP, and UEMP: untreated, enzymatically treated, ultrasound-treated, and ultrasound- and enzyme-treated myofibrillar protein without heat treatment, respectively; HMP, HEMP, HUMP, and HUEMP: corresponding MPs with heat treatment.

MP is composed of multiple proteins, including myosin, C-protein (120–140 kDa), actin (45 kDa), α-actinin (90 kDa), tropomyosin (35–40 kDa), and troponin T (29 kDa). Myosin can be further divided into myosin heavy chain (MHC, 200 kDa) and myosin light chain (MLC, 17 and 25 kDa) based on molecular weight. As shown in Figure 3A, the bands for myosin and actin were significantly darker after heat treatment. In the UMP and HUMP groups, there was an increase in polymers at the top of the stacking gel, with darker band colors and almost no change in their distribution. However, the high-molecular-weight bands in EMP almost disappeared, and similar changes were observed in the bands of MP after UGT and the corresponding heat treatment. This may be due to the significant reduction in the molecular weight of myofibrillar protein to 15 kDa or lower after enzymatic treatment. Li et al. [44] found that ultrasound promoted Qingke protein aggregation, resulting in increased polymers at the top of the stacking gel and significantly weakened protein bands. The bands became significantly lighter after TGase and ultrasound-assisted TGase treatment. Liu et al. [45] also found that ultrasound treatment can increase the molecular weight of some MP molecules, causing them to remain in the stacking gel. The darker band colors in HMP may be due to the unfolding of the MP structure influenced by heat treatment and ultrasonic cavitation, exposing more internal hydrophobic groups that can bind to Coomassie Brilliant Blue staining solution and reducing the molecular weight of MP, thereby promoting its dissolution in low-salt PBS and allowing more stained and separable proteins to enter the corresponding molecular weight range on the gel. Additionally, the electrophoresis pattern showed that ultrasound significantly degraded myosin heavy chains but had little effect on small-molecular-weight proteins, such as actin and tropomyosin. These results are consistent with the previously mentioned research findings on the hydrophobicity of modified MP.

#### 3.3.2. FTIR

A previous study established that the absorption peak in the amide A band region (3700–3200 cm^−1^) is primarily associated with N–H and O–H stretching vibrations, whereas the amide I band (1600–1700 cm^−1^) is attributed to C=O stretching, C–N stretching, and N–H in-plane bending [46]. As illustrated in Figure 3B, the spectra of the modified MPs closely resembled those of the untreated control, suggesting that ultrasound, enzymatic modification, UGT, and thermal processing did not generate new chemical bonds. However, pronounced red shifts in the amide A band were detected in the UMP, HMP, and HUMP groups, indicating that ultrasound and heat treatment altered the N–H stretching vibrations and consequently strengthened the intermolecular protein interactions [47]. In contrast, the EMP, HEMP, and UEMP groups exhibited blue shifts, with peak positions shifting from 3420.6 to 3441.1, 3435.2, and 3431.3 cm^−1^, respectively. Such spectral changes are likely attributable to transglutaminase-induced myofibrillar dissociation, which perturbs non-covalent interactions involving N–H or O–H groups, leading to bond shortening and an increase in local electron density [30]. Moreover, compared to the control, all treatment groups showed reduced transmittance in the amide I region. This reduction likely reflects the enhanced exposure of hydrophobic residues and concomitant alterations in the secondary structure induced by the modification [41]. These observations are consistent with the increased surface hydrophobicity of MPs, providing spectroscopic evidence linking structural rearrangements to protein functionality.

#### 3.3.3. Endogenous Fluorescence Spectroscopy

Tryptophan in proteins is used as an indicator to detect changes in the tertiary structure of proteins because of its extreme sensitivity to environmental variations. As shown in Figure 3C, after ultrasonic treatment, the fluorescence spectrum of the MP shifted towards longer wavelengths, and the fluorescence intensity decreased. This indicates that ultrasound facilitates the unfolding of the MP structure, increasing the polarity of the environment in which the internal hydrophobic groups are located. Similarly, a decrease in fluorescence intensity was observed after ultrasonic treatment of grouped MP [48]. Additionally, after enzymatic treatment and UGT, the fluorescence intensity further decreased, and a bimodal peak appeared; however, no trend was observed in the spectral shift. Fu et al. [49] reported an increase in fluorescence intensity after enzymatic treatment. The difference in results is speculated to be due to variations in salt or MP type, which lead to differences in protein structure [50]. Furthermore, as shown in Figure 3D, the fluorescence intensity of the MP significantly increased after heat treatment, and the wavelength of the maximum absorption peak exhibited a red shift after heat treatment. Similarly, compared with the MP group, the fluorescence intensity slightly increased in the HEMP and HUEMP groups. The increase in fluorescence intensity may be due to the more aggregated structure of MP after heat treatment, causing some hydrophobic groups to be deeply embedded within the protein in a nonpolar environment and thus displaying higher fluorescence intensity. Additionally, in the HUMP group, the fluorescence intensity decreased, and the wavelength exhibited a slight red shift. This may be because heat treatment after ultrasound further unfolds the MP, exposing tryptophan residues to a polar environment [51].

### 3.4. Rheological Properties

**Figure 4:**
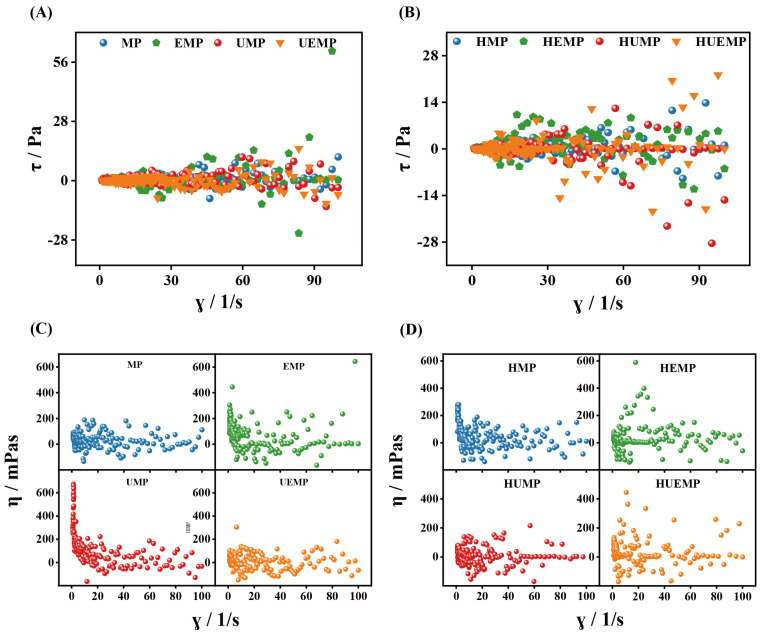
Rheological properties of different samples: (**A**) Shear stress of unheated MP, (**B**) Shear stress of heated MP, (**C**) Apparent viscosity of unheated MP, and (**D**) Apparent viscosity of heated MP. MP, EMP, UMP, and UEMP: untreated, enzymatically treated, ultrasound-treated, and ultrasound- and enzyme-treated myofibrillar protein without heat treatment, respectively; HMP, HEMP, HUMP, and HUEMP: corresponding MPs with heat treatment.

Viscosity, a measure of a fluid’s resistance to flow, is primarily governed by the intermolecular interactions among protein chains [20]. As illustrated in Figure 4A,B, the shear stress-shear rate curves of the MP solutions before and after treatment exhibited nonlinear behaviors, confirming that all samples behaved as non-Newtonian fluids. As shown in Figure 4C,D, the zero-shear viscosity of untreated MP ranged from 43.72 to 78.83 mPa·s. In contrast, ultrasonic-assisted enzymatic treatment narrowed this distribution (21.22–53.95 mPa·s) and reduced the overall viscosity. This reduction is likely attributable to the combined effects of ultrasonic cavitation and enzymatic deamidization, which disrupts protein–protein interactions and consequently suppresses aggregation and network formation [52]. Moreover, with increasing shear rate, the apparent viscosity of all samples progressively decreased to near zero, demonstrating shear-thinning behavior [53]. Following heat treatment, the viscosity of HUEMP remained consistently lower than that of HMP. Specifically, the zero-shear viscosity values ranged from 81.66 to 272.3 mPa·s for HMP and from 8.5 to 112.5 mPa·s for HUEMP, suggesting that the viscosity-reducing effects of ultrasonic-enzymatic treatment were partially retained after heating. Nevertheless, compared with unheated samples, heat-treated MP solutions exhibited higher viscosity, suggesting that thermal processing enhances the stability of protein solutions [54]. The observed increase in viscosity may be ascribed to the promotion of intermolecular associations during heating, which reduced fluidity and shear responsiveness [55].

## 4. Conclusions

In this study, UGT markedly enhanced tilapia myofibrillar protein solubility under low-salt conditions. This treatment also substantially increased the surface hydrophobicity, total sulfhydryl group content, and turbidity while simultaneously decreasing the particle size, ζ-potential, and intrinsic fluorescence intensity. Fourier-transform infrared spectroscopy further revealed a blue shift in the amide A band and reduced transmittance of the amide I band, indicating partial unfolding and reorganization of the protein secondary structure. Following thermal treatment, the proteins displayed enhanced structural stability, as demonstrated by the increased surface hydrophobicity and fluorescence intensity, red shift in the amide A band, and elevated solution viscosity. Collectively, these findings suggest that UGT induces a moderately expanded yet stable conformation in myofibrillar proteins, providing a theoretical framework for the rational design and application of novel protein-based food systems.

## Figures and Tables

**Table 1:** Changes in Surface Hydrophobicity, Total Sulfhydryl Content, and Turbidity of Myofibrillar Protein Before and After Heat Treatment.

	Surface Hydrophobicity	Total Sulfhydryl Content	Turbidity
MP	267.00 ± 7.38 ^h^	101.40 ± 8.61 ^bc^	0.153 ± 0.008 ^c^
EMP	353.97 ± 2.69 ^d^	91.84 ± 10.27 ^c^	0.121 ± 0.009 ^de^
UMP	287.20 ± 7.76 ^g^	99.95 ± 6.33 ^bc^	0.19 ± 0.004 ^b^
UEMP	393.81 ± 6.51 ^c^	112.75 ± 4.64 ^abc^	0.444 ± 0.05 ^a^
HMP	322.32 ± 2.77 ^f^	127.92 ± 22.79 ^a^	0.132 ± 0.004 ^cd^
HEMP	431.80 ± 11.86 ^b^	120.93 ± 15.60 ^ab^	0.125 ± 0.006 ^cd^
HUMP	335.47 ± 7.20 ^e^	121.99 ± 4.93 ^ab^	0.092 ± 0.02 ^e^
HUEMP	472.96 ± 5.12 ^a^	129.88 ± 20.47 ^a^	0.111 ± 0.01 ^de^

Note: Lowercase letters indicate significant differences between the treatments.

## Data Availability

The original contributions presented in the study are included in the article. Further inquiries can be directed to the corresponding authors.
